# An Abnormal Inflammatory Pattern Associated with Long-Term Non-Progression of HIV Infection Impacts Negatively on Bone Quality

**DOI:** 10.3390/jcm11102927

**Published:** 2022-05-22

**Authors:** Jade Soldado-Folgado, Juan José Chillarón, Esperanza Cañas-Ruano, Itziar Arrieta-Aldea, Alicia González-Mena, Fabiola Blasco-Hernando, Hernando Knobel, Natalia Garcia-Giralt, Robert Güerri-Fernández

**Affiliations:** 1 Department of Internal Medicine, Hospital del Mar Medical Research Institute (IMIM), 08003 Barcelona, Spain; jsoldado@psmar.cat; 2 Departament de Medicina, Universitat Autònoma de Barcelona, 08193 Barcelona, Spain; 3 Department of Endocrinology, Hospital del Mar Medical Research Institute (IMIM), 08003 Barcelona, Spain; jchillaron@psmar.cat; 4 Department of Infectious Diseases, Hospital del Mar Medical Research Institute (IMIM), 08003 Barcelona, Spain; mariadelaesperanza.canas.ruano@psmar.cat (E.C.-R.); iarrieta@psmar.cat (I.A.-A.); agonzalez@psmar.cat (A.G.-M.); fblasco@psmar.cat (F.B.-H.); hknobel@psmar.cat (H.K.); ngarcia@imim.es (N.G.-G.); 5 Department of Medicine and Life Sciences (MELIS), University Pompeu Fabra, 08002 Barcelona, Spain; 6 Instituto de Salud Carlos III, Centro de Investigación Biomédica en Red en Enfermedades Infecciosas (CIBERINFEC), 28029 Madrid, Spain

**Keywords:** HIV, bone metabolism, microindentation, inflammation, immunoactivation, cytokines

## Abstract

Introduction. Long-term non-progressors (LTNPs) are HIV-infected individuals (HIV^+^) whose viral replication is controlled. However, these individuals experience complications associated with HIV, among them, bone remodeling impairment. This study aims to perform a comprehensive bone health assessment and its association with the inflammatory status of HIV^+^ LTNPs. A cross-sectional study was conducted comparing bone strength components (bone mineral density and bone tissue quality) between age-, sex-, and comorbidities-matched groups of HIV^+^ LTNPs, HIV^+^ progressors, and HIV-negative individuals. A panel of bone turnover and inflammatory biomarkers was measured in fasting plasma using ELISA. Bone tissue quality was assessed by bone microindentation, a technique that directly measures the bone resistance to fracture and yields a dimensionless quantifiable parameter called bone material strength (BMSi). Thirty patients were included: ten LTNPs, ten HIV^+^ progressors, and ten HIV-negative individuals. LTNPs showed an abnormal pattern of immune activation that was represented by significantly lower levels of anti-inflammatory cytokine IL-10 (*p* = 0.03), pro-inflammatory cytokine IL-8 (*p* = 0.01), and TNF-α (*p* < 0.001) with respect to the other groups. Regarding bone health, LTNPs presented lower BMSi, and thus, worse bone tissue quality than HIV-negative individuals (83 (78–85) vs. 90 (89–93), respectively; *p* = 0.003), and also lower BMSi than HIV^+^ progressors (83 (78–85) vs. 86 (85–89), respectively; *p* = 0.022). A trend was found of lower BMSi in HIV^+^ progressors with respect to the HIV-negative individuals (86 (85–89) vs. 90 (89–93), respectively; *p* = 0.083). No differences were detected in bone mineral density between groups. In conclusion, LTNPs showed a different inflammatory profile, along with worse bone tissue quality, when compared to HIV^+^ progressors and HIV-negative individuals. This may contribute to increasing evidence that HIV infection itself has a deleterious effect on bone tissue, likely through a persistent altered inflammation status.

## 1. Introduction

Since HIV survival has improved, the prevalence of age-related illnesses related to chronic HIV infection has also increased [1]. Among them, low bone mineral density (BMD) and the increased risk of fracture observed in HIV-infected patients [2,3] have become comorbidities of concern and have been broadly studied.

The impact of HIV on bone mineral density is well-characterized. Despite the fact that multiple factors appear to be involved, the relative contribution of HIV infection to the development of low BMD is being elucidated. In addition to the classical risk factors, an implication not only of HIV itself, but also of antiretroviral therapy (ART), has been suggested [4,5]. A study with the HIV UPBEAT cohort concluded that HIV infection itself was directly associated with lower BMD, and this was mediated by alterations in bone metabolism [6]. Recent data suggest that immune activation, even among virologically suppressed individuals, may contribute to bone loss [7]. Long-term non-progressors (LTNPs), a group of HIV-infected individuals that spontaneously control the infection, may present an ideal scenario to study the potential role of viral persistence, secondary inflammation and their impact on bone metabolism. 

The gold standard for the study of bone mass is the Dual Energy X-ray Absorptiometry (DEXA) scan, which measures BMD and correlates it with fracture risk. DEXA is a diagnostic tool widely used for HIV individuals. However, other components of bone strength, such as microarchitecture or bone tissue quality, have been demonstrated topics of interest in the study of bone health [8,9,10]. Bone microindentation testing (BMT) is a complementary technique to DEXA [11] that is being extensively studied. This is a minimally invasive technique that can directly analyze bone tissue quality [12], a different component of bone strength. Previous studies demonstrated significant differences between bone tissue quality and bone mineral density in the HIV population [8,10], but information about bone tissue quality in LTNPs remains to be unveiled. 

**Figure 1:**
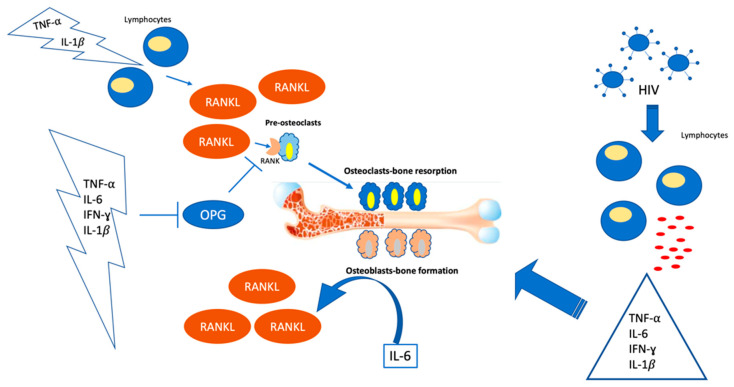
Schematic representation of how the HIV virus infects and activates lymphocytes that increase the production of different inflammatory cytokines (IL-6, TNF-α, IFN-γ). These cytokines, in turn, stimulate the maturation of the osteoclast, the cell responsible for bone resorption through the RANK/RANKL/OPG pathway. The source of RANKL is different cells, such as lymphocytes or osteoblasts, in response to inflammatory cytokines. HIV: Human Immunodeficiency Virus; RANK: Receptor activator of nuclear factor κ B; RANKL: Receptor activator of nuclear factor κ B-Ligand; OPG: Osteoprotegerin.

Chronic inflammatory illnesses, such as HIV infection, disrupt bone metabolism and are frequently linked to the development of osteoporosis. Bone remodeling is a complex process that can be disrupted by persistent inflammation, leading to increased bone loss. In this condition, immune system mediators, such as the pro-inflammatory cytokines tumor necrosis factor-alpha (TNF-α), interleukin-1beta (IL-1), IL-6, or interferon-γ, govern bone loss. HIV alters the immune response and the amounts of certain cytokines, which can have a detrimental influence on bone. These inflammatory mediators have a direct impact on bone regulating pathways, such as RANK (Receptor activator of nuclear factor κ B )/RANKL (Receptor activator of nuclear factor κ B Ligand)/OPG (Osteoprotegerin). Cytokines may function by promoting osteoclast activity, resulting in increased bone loss. TNF and IL-1 enhance mature osteoclast activity by boosting systemic RANKL production through lymphocytes and endothelial cells [13], while IL-1 acts on osteoblasts to generate PGE2 synthesis, both indirectly encouraging osteoclast development. Other immune mediators, such as IL-6, have been linked to joint erosion in rheumatoid arthritis patients, implying that they play a role in bone resorption [14]. IL-6 operates on osteoblasts and T-lymphocytes to enhance RANKL synthesis, similar to other inflammatory cytokines [15] (Figure 1).

Taking all this into account, the aim of this study is to analyze bone tissue quality measured by bone microindentation testing in LTNPs compared to HIV^+^ progressors and HIV-negative individuals. As secondary objectives, we aimed to elucidate the molecular mechanisms involved in the bone impairment in LTNPs by analyzing the inflammation status, bone turnover markers, and bone signaling pathways. 

## 2. Patients and Methods

A cross-sectional 1:1:1 study was performed from June 2017 to February 2022. A group of LTNPs and another group of gender-, age-, comorbidities-, and body mass index (BMI)-matched ART-naïve HIV individuals (HIV^+^ progressors) were selected from the Infectious Diseases Department at Hospital del Mar (Barcelona, Spain). A gender-, age-, comorbidities-, and BMI-matched HIV-negative control group was recruited from a Primary Care Center in Barcelona. 

The LTNP group was defined as patients without previous antiretroviral therapy (ART) and plasma HIV RNA levels below 500 copies/mL for the last 24 months [16]. The HIV^+^ progressor group was defined as patients without previous ART, plasma HIV RNA levels > 500 copies/mL, and at least 12 months since the diagnosis of the infection. 

Exclusion criteria were Hepatitis C or Hepatitis B co-infection and any disease or treatment that can affect bone metabolism. In the case of women, we did not include post-menopausal women due to the known impact of menopause on bone quality and bone mineral density. 

All patients underwent a fasting blood test for the evaluation of renal function, calcium, phosphate, and bone biomarkers, such amino pro-peptide of type 1 collagen-(P1NP), collagen type I cross-linked C telopeptide (CTX), parathormone (PTH), serum 25-hydroxy-vitamin D, and bone alkaline phosphatase (BAP), by immunoenzymatic electrochemiluminescence, ECLIA (Roche Diagnostics, Indianapolis, IN, US). DKK1, osteoprotegerin (OPG), and sclerostin (SOST) were determined by enzyme-immunosorbent multiassay panel (Merk Millipore multiassay ELISA, Munich, EU). Inflammatory and procoagulation markers, such as high-sensitivity C-reactive protein (CRP), erythrocyte sedimentation rate (ESR), fibrinogen, and D-dimer, were analyzed by ELISA (Roche Diagnostics, Indianapolis, IN, USA) according to the manufacturer’s instructions.

Bone mineral density (BMD) was measured by DEXA with a Hologic QDR 4500 SR Bone densitometer (Hologic, Inc., Waltham, MA, USA) in the lumbar spine (LS) and femoral neck (FN) of all participants. 

Bone microindentation was performed using an OsteoProbe Reference Point Indenter (ActiveLife Inc., Santa Barbara, CA, USA), by a single trained operator. The procedure has been previously reported [9]. In brief, after skin disinfection and local anesthesia, bone microindentation is performed over the midshaft tibiae cortical bone, and the results are normalized to a polymethylmethacrylate phantom. Bone microindentation yields a dimensionless quantifiable parameter called bone material strength index (BMSi), which is positively correlated with bone tissue quality [9,10]. All measurements of BMSi were included, and the mean value was considered as the outcome. 

This study was approved by the local Clinical Research Ethics Committee, and all participants gave written informed consent (Ethics Committee number 2013/5250/I).

### Statistical Analysis

Continuous measures are described by medians and interquartile ranges (IQR), and categorical variables are described with frequencies and percentages. 

Non-normally distributed variables were tested at baseline by Mann–Whitney U. The LTNP group was the reference group for the comparisons. 

The Kruskal–Wallis test was used for the comparison of bone strength parameters, inflammatory markers, and bone turnover markers among the three groups. A post hoc statistical analysis was performed using Dunn’s test as the appropriate nonparametric pairwise multiple-comparison procedure. 

To estimate the sample size required to detect differences between LTNPs and HIV^+^ progressors, we considered previously published differences in bone quality between ART-naïve HIV individuals and non-HIV individuals, and also between treated undetectable HIV patients [9,10,17]. These last two groups represented individuals in whom the differences were expected to be smaller. We accepted an alpha risk of 0.05 and a beta risk of 0.2 in a two-sided test and estimated that eight subjects were necessary in the LTNP group and eight in the HIV^+^ progressor group to recognize as statistically significant a difference greater than or equal to 6 BMSi units. The common standard deviation is assumed to be 4. However, due to the reduced sample size and the fact that three groups were included, we performed a post hoc potency calculation with the BMSi values obtained. 

For the statistical analysis, STATA v14.02 was used (College Station, TX, USA).

## 3. Results

### 3.1. Baseline Characteristics

Thirty patients were included: ten LTNPs, ten HIV^+^ progressors, and ten HIV-negative individuals (controls). The main baseline characteristics are shown in Table 1. No differences in the CD4 + T-cell count were found between the HIV groups: 477 (393–730) in LTNPs vs. 510 (341–579) in HIV^+^ progressors (*p* = 0.624), despite LTNPs having a longer duration of infection, with 6 years since diagnosis (3–13), compared to 3 years (2–4) in HIV^+^ progressors (*p* = 0.02) and a significantly lower viral load (66 copies/mL (39–145) in LTNPs and 13,180 copies/mL (4396–160,977) in HIV^+^ progressors; *p* < 0.001). 

### 3.2. Post Hoc Power Calculation

Results from the post hoc power calculation provided a power of 95.5% for the variable BMSi basal. This was with a balanced design (ten patients per group), sample means equal to 90.2, 81.9 and 87.2 for non-HIV, non-progressor HIV, and HIV progressors, respectively, and a within-groups mean square error of 17.75.

### 3.3. Bone Strength Components

#### 3.3.1. Bone Mineral Density, In Vivo Microindentation (Bone Quality)

BMSi, lumbar spine BMD, and femoral neck BMD values are presented in Table 2 for each group of patients. No differences were found in the BMD at any measured site among groups.

**Figure 2:**
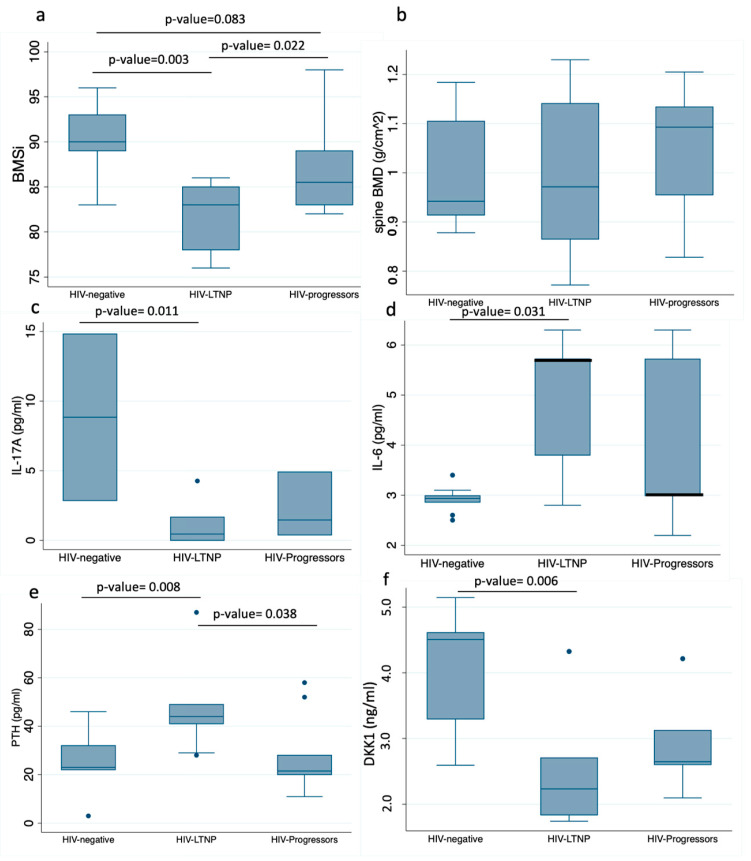
Comparison of different elements of bone strength, inflammation, and bone markers between groups. Bone strength components: (**a**) BMSi: bone tissue quality; (**b**) spine BMD. Inflammatory markers: (**c**) IL-17A; (**d**) IL-6. Bone metabolism markers: (**e**) PTH; (**f**) DKK1. Lines with *p*-value show the post hoc analysis between groups. Box-plot shows median and IQR. Filled dots (circles) show outlier values.

The LTNP group presented lower BMSi, and thus, worse bone tissue quality than HIV-negative individuals (median (IQR)): (83 (78–85) vs. 90 (89–93), respectively; *p* = 0.003). They also presented lower BMSi than the HIV^+^ progressor group (83 (78–85) vs. 86 (85–89), respectively; *p* = 0.022). Likewise, there was a trend of lower BMSi in the HIV^+^ progressor group with respect to the HIV-negative group (86 (85–89) vs. 90 (89–93, respectively; *p* = 0.083) (Figure 2). 

#### 3.3.2. Bone Turnover Markers

When comparing different bone turnover markers, DKK1 was significantly lower in HIV^+^ individuals, with the worst value in the LTNP group (control group vs. HIV^+^ LTNPs: *p* = 0.006). No differences were found between HIV^+^ groups (HIV^+^ progressors vs. HIV^+^ LTNPs; *p* = 0.475). In contrast, PTH was increased in HIV^+^ LTNPs with respect to the HIV-negative group (*p* = 0.008) and with respect to HIV^+^ progressors (*p* = 0.038). No differences were found in other bone turnover markers (Table 2). 

### 3.4. Inflammatory Markers

LTNPs showed an abnormal pattern of immune activation that was represented by significantly lower levels of anti-inflammatory cytokine IL-10 (*p* = 0.03), pro-inflammatory cytokine IL-8 (*p* = 0.01), and TNF-α (*p* < 0.001) with respect to the other groups (Table 2). The LTNP group had also lower levels of IL-17A compared to HIV-negative individuals (*p* = 0.011) and a trend of lower levels compared to HIV^+^ progressors (*p* = 0.08). In contrast, LTNPs had significantly increased levels of pro-inflammatory IL-6 cytokine compared to HIV-negative individuals (*p* = 0.031) and a trend of higher levels of IL-6 compared to HIV^+^ progressors (*p* = 0.08).

## 4. Discussion

We report a significant impact of HIV infection on bone tissue quality in HIV long- term non-progressors, despite having no differences in bone mineral density, along with an abnormal inflammatory pattern compared to non-infected individuals. Long-term non-progressors are a small subset of HIV-infected individuals on the order of 0.2 to 3.34% [1,18] who have been the focus of scientific interest due to their ability to suppress HIV replication without treatment. Despite naturally controlling the infection, many studies have found that LTNPs had worse clinical outcomes in terms of cardiovascular diseases, non-AIDS-defining infections, psychiatric illness, and increased rates of all-cause hospitalization [1]. However, to our knowledge, there is a lack of evidence about bone health and bone remodeling factors in this group [1]. 

We have studied different components of bone strength. Firstly, we examined the amount of mineral component in the bones, measured as bone mineral density; no differences among the groups were detected. Although the small sample size of the groups could have an impact, it must be acknowledged that the groups were well-balanced and comparable. In prior studies, some differences in BMD have been found between HIV^+^ and non-HIV individuals [4,19]. However, since newly diagnosed HIV individuals in our area are mostly healthy individuals with no exposure to classical risk factors for bone impairment (such as opioid abuse, malnutrition, tobacco, etc.), these differences were not detected in a prior larger publication on our cohort [9]. Secondly, the bone tissue quality measured by microindentation showed worse material properties in LTNPs with respect to both HIV-negative and HIV^+^ progressor individuals. The different behaviors of bone strength components in HIV individuals have previously been reported by our group [9,10]. Bone tissue quality is not only determined by the amount of mineral component, but also by the three-dimensional structure of the collagen and non-collagen protein matrix. Hence, processes that can influence the composition of these proteins could have an effect on bone structure and resistance and, thereby, increase fracture risk [20]. 

We previously reported a correlation between the inflammatory status associated with HIV infection and worse bone tissue quality [20]. In the present study, we report an immune disbalance in LTNPs, with a particular cytokine signature compared to healthy individuals but also to HIV^+^ progressors. Despite the small sample size of this study, this is biologically plausible due to the persistence of a non-replicating virus along with the immune changes. At the end, this disbalance could have a greater impact on the bone organic matrix than on the mineral component, leading to the lower BMSi and thus, worse bone tissue quality, found in LTNPs [8]. It is noteworthy that LTNP individuals had significantly longer exposition to HIV and no exposure to ART. This is because of the fact that, in the past, treatment was delayed among LTNPs until an increase in viral replication was detected. We entertain the hypothesis that these individuals suffered continuous exposure to the non-replicating virus and the consequent immune and inflammatory changes that may affect bone remodeling, thereby impairing the bone matrix composition and structure [17]. 

The increased risk of fractures associated with persistent inflammation has been previously observed in other pathologies with a high rate of immune activation. Sun et al. [21] observed upregulated levels of high-sensitivity C-reactive protein (hsCRP), TNF-α, IL-1β, IL-6, IFN-c-inducible protein 10 (IP-10), and RANTES in type 2 diabetes mellitus patients with bone fracture. We hypothesize that HIV infection in LTNPs would induce a loss in the immune balance between pro- and anti-inflammatory cytokines, which would impact negatively on bone quality measured by microindentation, as observed in other diseases such as diabetes [21]. Moreover, in some cases, ART has been deferred in LTNP individuals. However, according to these results, chronic low viral replication may have some deleterious effects on bone and likely on other organs, and early initiation of ART should be considered. 

Interestingly, we found that LTNPs had significantly lower IL-17A levels, corresponding to a prior report by our group where we found a positive correlation between IL-17A and bone tissue quality [20]. Despite the fact that the role of IL-17A in bone remains controversial, some studies suggest that IL17A inhibits the differentiation and activity of osteoclasts facilitating bone formation [22]. Thus, higher IL-17 could be related to better bone material properties through the inhibition of osteoclastogenesis and the promotion of bone repair through osteogenesis [23]. Otherwise, we can speculate that lower IL-17A levels could have the contrary effect. Moreover, IL17A-producing T-cells (Th17) are a subset of CD4+ T-cells, which maintain the gut epithelial barrier and prevent microbial translocation and are the target of HIV, since their number and functions are depleted in progressive HIV infection. It has been previously reported that ART quickly increases Th17 numbers and restores, partially, their polyfunctional capacity [24,25]. Hence, LTNP individuals could have their Th17 permanently reduced, and this could promote an impaired immune profile [26] affecting several tissues, such as bone. 

The Dickkopf Wnt Signaling Pathway Inhgibitor-1 (DKK1) binds to the LRP6 co-receptor and inhibits beta-catenin-dependent Wnt signaling. It is well-known that Wnt signaling is a master regulator of bone formation [27] and may be altered by persistent inflammation, mainly through DKK1 [28,29]. We found that LTNP individuals (and HIV^+^ progressors in less significance) presented decreased DKK1 levels compared to HIV-negative individuals. This may suggest a potential mechanism involved in the impaired bone tissue quality mediated by an altered inflammatory state in HIV patients through the Wnt/β-catenin pathway [27]. Decreased levels of DKK1 are found to be related to a reduced BMSi parameter rather than to BMD. Hence, the bone–immune interface could be regulated by the Wnt signaling pathway, where DKK1 would play a major role in bone quality.

Interestingly, we also found significantly increased levels of PTH in LTNPs, suggesting a close coupling between the immune system and PTH. It has been previously reported that systemic levels of IL-6 are regulated, among others, by PTH [30]. This may represent another mechanism by which long-term immune impairment would affect the quality of bone tissue. 

This study has some limitations that must be stated, the first being the limited sample size, mainly due to the low prevalence of LTNP individuals. This fact could make it difficult to detect some differences between groups. It is noteworthy that the groups in the study were balanced, and the baseline parameters were matched between groups. Of note, the obtained results are biologically plausible and open new areas of study in bone disease among HIV patients. Another limitation is the use of microindentation for bone strength evaluation; although it is a validated technique, still few centers have wide experience with it. However, our research group has great expertise in the use of this technique, which can contribute to the understanding of bone behavior under HIV infection. Moreover, the results from this study are in the same line with previous publications on HIV individuals, suggesting that HIV infection impacts negatively on bone health. Precisely, the HIV-associated abnormal inflammatory pattern reported in this study may show how HIV-induced chronic inflammation could produce deleterious effects on bone quality [20]. Further studies with other HIV cohorts are needed to corroborate these findings. Of special interest would be to study the group of elite controllers that spontaneously control viral replication [31].

## 5. Conclusions

In summary, we have found that LTNPs had significantly worse bone tissue quality, measured by in vivo microindentation, than non-infected individuals and HIV^+^ progressors. Chronic persistence of HIV, replicating or not, seems to be the driver of immune changes that lead to an altered inflammatory status indirectly affecting bone health. This finding adds new evidence to the potential deleterious effect of HIV infection on bone tissue. The association between the altered inflammatory status and bone tissue quality seems to be in the same direction as reported in prior publications. Moreover, the specific immune signature found in LTNPs seems to be associated with a worse bone tissue quality, likely through the Wnt pathway. Hence, this Wnt signaling could be a potential pharmacological target for protecting bone tissue in these patients. Further studies are needed to confirm these results and to prospectively assess the clinical significance of the findings reported in this work. 

## Figures and Tables

**Table 1:** Baseline characteristics of the HIV-negative individuals (control), HIV^+^ LTNP group, and HIV^+^ progressors. Main demographic, medical, and HIV characteristics of the three groups are included. *p*-value from the Kruskal–Wallis test. Quantitative variables shown as median (interquartile range).

	Control	HIV^+^ LTNP	HIV^+^ Progressors	*p*-Value
N	10	10	10	
Age (years)	42 (35–55)	47 (45–54)	44 (41–45)	0.413
Male (*n*, %)	5 (50%)	6 (60%)	7 (70%)	0.384
BMI (kg/m^2^)	24 (23–26)	25 (23–26)	24 (22–24)	0.125
Smoking (*n*, %)	5 (50%)	6 (60%)	6 (60%)	0.548
Alcohol (>10 g/d) (*n*, %)	0	1 (10%)	0	0.833
Ex-IDU (*n*, %)	0	1 (10%)	0	0.833
Recreational drugs (*n*, %)	2 (20%)	2 (20%)	3 (30%)	0.428
Previous fracture (*n*, %)	0	0	0	
Family history of fracture (*n*, %)	0	1 (10%)	1 (10%)	0.441
Prevalent spine fractures (*n*, %)	0	0	0	
eGFR < 60 mL/min	0	0	0	
eGFR (CKD-EPI (mL/minl)	82 (78–87)	85 (77–89)	85 (78–91)	0.188
Years since HIV diagnosis	0	6 (3–13)	3 (2–4) ^a^	0.032
Nadir CD4 count (per mL)		438 (305–678)	417 (341–548)	0.939
Current CD4 count (per mL)		444 (393–730)	510 (341–579)	0.791
Current viral load (per mL)		66 (39–145)	13,180 (4396–160,977) ^a^	0.021
Ever met AIDS criteria (*n*, %)		0	1 (10%)	

^a^. *p*-value<0.05 HIV^+^ LTNP vs. HIV^+^ Progressors. BMI: Body Mass Index; IDU: Intravenous Drugs Users; eGFR: estimated Glomerular Filtration Rates; CKD-EPI: Chronic Kidney Disease Epidemiology Collaboration.

**Table 2:** Bone strength components, inflammation, and bone turnover markers among groups. Comparison between three groups of study (Control (HIV-negative), HIV^+^ LTNP, and HIV^+^ progressors). *p*-value from the Kruskal–Wallis test. Results are shown as median (IQR), unless indicated otherwise.

	Control		HIV^+^ LTNP		HIV^+^ Progressors		*p*-Value
Bone Strength							
BMSi	90	(89–93)	**83**	**(78–85) ^1,2^**	**86**	(83–89) *	0.001
Lumbar spine BMD (g/cm^2^)	0.936	(0.91–0.988)	0.933	(0.86–1.14)	1.05	(0.95–1.13)	0.558
Femoral neck BMD (g/cm^2^)	0.774	(0.73–0.82)	0.821	(0.787–0.98)	0.848	(0.79–0.87)	0.104
Inflammation							
hs-CRP (mg/dl)	0.14	(0.002–0.47)	0.15	(0.09–0.32)	0.39	(0.15–0.78)	0.182
D-Dimer (IU/mL)	190	(143–191)	240	(147–290)	180	(131–202)	0.389
IFN-γ (pg/mL)	11.7	(10.4–11.9)	9.85	(0.46–24.31)	8.35	(6.5–12.7)	0.613
IL-10 (pg/mL)	3.41	(3.24–3.57)	**2.85**	**(0.22–2.85) ^1,2^**	3.5	(3.24–3.57)	0.023
IL-17A (pg/mL)	8.83	(2.85–14.82)	**0.46**	(0.46–1.67) ^1,^**	1.46	(0.39–4.91)	0.014
IL-2 (pg/mL)	2.41	(0.58–3.14)	1.46	(0.5–1.46)	2.41	(1.46–2.41)	0.554
IL-6 (pg/mL)	2.92	(2.86–2.99)	**5.72**	(3.8–5.72) ^1,^**	2.99	(2.99–5.72)	0.231
IL-8 (pg/mL)	48.2	(35.6–51.2)	**1.89**	**(0.97–5.85) ^1,2^**	14.4	(8.27–37.2)	0.004
Soluble CD40 Ligand (ng/mL)	7.26	(5.1–7.5)	9.3	(6.2–17.7)	5.5	(4–10.2)	0.341
TNF-α (pg/mL)	16.9	(11.3–24.51)	**3.7**	**(2.07–4.69) ^1,2^**	18.3	(16.7–22.02)	0.004
IL-6 Soluble Receptor (ng/mL)	28.6	(22.9–37.2)	32.6	(22.7–33.6)	29.7	(25.9–33.1)	0.915
Bone Metabolism Markers							
DKK1 (ng/mL)	4.5	(3.2–4.6)	**2.2**	**(1.8–2.7) ^1^**	2.6	(2.6–3.1)	0.043
OPG (ng/mL)	0.5	(0.4–0.6)	0.53	(0.46–0.61)	0.5	(0.44–0.52)	0.678
SOST (ng/mL)	1.6	(1.3–1.6)	1.2	(0.3–1.5)	1.4	(1.1–1.5)	0.417
P1NP (ng/mL)	30.1	(22.3–36)	31.4	(28.8–48.9)	43.5	(34.4–52.2)	0.231
Ctx (ng/mL)	0.21	(0.11–0.47)	0.31	(0.19–0.44)	0.25	(0.24–0.35)	0.678
Bone Alkaline Phosphatase (µg/mL)	12.5	(7.5–17.8)	10.8	(8.8–14.7)	13.3	(11.7–19.1)	0.791
25OH Vitamin D (ng/mL)	27	(22.8–41.2)	26.6	(23–43)	25.1	(13.4–29.1)	0.544
PTH (pg/mL)	23	(22–32)	**44**	**(41–49) ^1,2^**	21	(20–28)	0.013

BMSi: Bone Material Strength index; BMD: Bone Material Density;hs-CRP: high-sensitivity C-reactive protein; OPG: Osteoprotegerina; SOST: Sclerostin. **Shown in Bold**: ^1^ corresponds to *p*-value < 0.05 compared to HIV-negative individuals (controls). ^2^ corresponds to a *p*-value < 0.05 obtained after comparison to HIV+ non-controllers. * *p* = 0.08 with respect to controls. ** *p* = 0.08 with respect to HIV^+^ non-controllers.

## Data Availability

Some or all datasets generated and/or analyzed during the current study are not publicly available but could be available from the corresponding author on reasonable request.
